# Health-related quality of life in patients with peripheral nerve tumors: results from the German multicentric Peripheral Nerve Tumor Registry

**DOI:** 10.3389/fonc.2024.1398252

**Published:** 2024-04-22

**Authors:** Nadja Grübel, Gregor Antoniadis, Uerschels AK, Benjamin Mayer, Ralph König, Christian Rainer Wirtz, Andrej Pala, Nora F. Dengler, Maria Teresa Pedro

**Affiliations:** ^1^ Peripheral Nerve Unit, Department of Neurosurgery, University Medicine of Ulm, Günzburg, Germany; ^2^ Department of Neurosurgery, University Medicine Essen, Essen, Germany; ^3^ Institute of Epidemiology and Medical Biometry, University of Ulm, Ulm, Germany; ^4^ Department of Neurosurgery, University Medicine Charité Berlin, Berlin, Germany

**Keywords:** peripheral nerve tumor, life quality, pain, malignant peripheral nerve tumor, neurofibromatosis

## Abstract

**Objective:**

Peripheral nerve tumors (PNTs) are rare diseases. So far, no multicenter data on diagnostics, the efficacy of treatment, long-term outcomes, and health-related quality of life (HRQoL) exist. The establishment of the Peripheral Nerve Tumor Registry (PNTR) in 2015 allows for the systematic analysis of patients with tumors associated with peripheral nerves. The present study aims to investigate the impact of PNT on an individual’s HRQoL and the effect of surgery.

**Methods:**

HRQoL was pre- and postoperatively assessed by the Euro-Qol-5D-5L (EQ-5D-5L) and Euro-Qol visual analog scale (EQ-VAS) survey in the retrospective and prospective study arm in three active participating study centers. An index was calculated based on the EQ-5D-5L for the quantification of health state (0: worst possible state of health, 1: best possible state of health). The EQ-VAS ranges from 0% (worst imaginable health status) to 100% (best possible health status). Patient characteristics (age, sex), as well as disease (histopathological entity) and treatment (pre- and postoperative symptoms, type of treatment)-specific data, were analyzed.

**Results:**

Data from 171 patients from three high-volume centers were included, with schwannoma (70.8%, *n* = 121) and neurofibroma (15.8%, *n* = 27) being the most prevalent histopathological diagnoses. Both the median health index value (preoperative: 0.887, *n* = 167; postoperative: 0.910, *n* = 166) and the median EQ-VAS (preoperative: 75%, *n* = 167; postoperative: 85%, *n* = 166) of the entire cohort regarding all histopathological diagnosis improved significantly after surgical therapy (*p* < 0.001). Preoperatively, 12.3% (*n* = 21) reached the highest index score of 1.0 in EQ-5D-5L and 100% in the EQ-VAS score in 5.3% (*n* = 9) of all patients. Postoperatively, the highest index score of 1.0 and 100% in the EQ-VAS score increased significantly and were achieved in 33.3% (*n* = 57) and 11.1% (*n* = 19) of the patients, respectively (*p* < 0.001).

**Conclusion:**

For the first time, our study presents multicenter data on life quality and the effect of surgery in primarily benign peripheral nerve tumors. Early surgery at a specialized center could improve neurological outcomes and, in conclusion, better QoL. In summary, surgical therapy significantly improved the entire cohort’s QoL, VAS, and analgesia.

## Introduction

Overall, peripheral nerve tumors (PNTs) are rare diseases, occurring frequently in the extremities, torso, or neck (1–3). The affected patients usually complain of pain, muscle weakness, or sensory deficits. Tumor size and the exact entity of PNT can vary substantially, leading to a large spectrum of therapeutic pathways, outcomes, and prognoses. Right from the start of treatment, the main challenge is to choose between open biopsy, surgical resection, or conservative management (3). To date, clinical trial evidence with robust epidemiological and clinical information is limited mainly to single-center results regarding schwannoma and neurofibroma, most frequent among PNTs and benign tumors (4). Other rare entities, such as perineurioma, amyloidoma, lipoma, desmoid, lymphoma, and malignant peripheral nerve sheath tumors (MPNSTs), are scarcely described (5, 6). No studies on life quality in patients with PNT have been conducted to date.

Quality of life is a complex concept combining the fields of medicine and public health to find a combined endpoint concerning diagnostic modalities, types of therapy, and life quality (7). Furthermore, the World Health Organization (WHO) described life quality as “An individual’s perception of their position in life in the context of the culture in which they live and about their goals, expectations, standards, and concerns” (8). According to the revised version of the “Declaration of Geneva” in 2017, it is within the responsibility of all doctors to restore health and not impair the overall well-being of patients and to implement health aspects to treatment and decision-making in everyday clinical practice (9).

Health-related quality of life is the subject of medical care and represents only a small but important part of overall quality of life. For example, HRQoL is essential for medical decision-making and predicts treatment success and overall survival (7).

This study aims to evaluate HRQoL in patients with tumors associated with the peripheral nerves and the effect of surgical therapy on these patients and their life quality.

## Methods

### Study design—Peripheral Nerve Tumor Registry

The establishment of the multicentric Peripheral Nerve Tumor Registry (PNTR) in 2015 in Germany allows for the systematic analysis of patients with benign, malignant, and other rare tumor entities associated with the peripheral nerves. So far, no multicentric data on peripheral nerve tumors exist in Europe. The PNTR was divided into a retrospective (2015–2016) and prospective (since 2017) study arm. Patient characteristics (age, sex) as well as disease (affected nerve, tumor location, histopathology), surgical treatment (type of treatment, pre- and postoperative symptoms), radiological imaging, diagnosis of neurofibromatosis (NF), data on health-related quality of life (HRQoL), return to work, and long-term follow-up data were analyzed. The long-term goal is to create uniform treatment recommendations (10).

This substudy of the PNTR contains a partially retrospective and prospective analysis of HRQoL in 171 patients with benign, malignant, and rare peripheral nerve tumors that were surgically treated in either the Department for Neurosurgery in Günzburg, University Hospital Ulm; the Department for Neurosurgery in Berlin, Charité University Hospital; or the Department for Neurosurgery in Essen, University Hospital, which were treated between January 2015 and January 2023. All patients gave written permission. This study was approved by our local ethics committees in Ulm (Nr. 249/17) and Berlin (EA4/058/17) and is registered with the German Trials Registry (www.drks.de) (10).

### Inclusion and exclusion criteria

All patients who were diagnosed with a tumor in association with a peripheral nerve and surgically treated in one of the high-volume recruiting study centers were enrolled.

### Assessment of the HRQoL

The assessment of HRQoL was conducted by the standardized preference-based questionnaire Euro-Qol-5D-5L (EQ-5D-5L) and Euro-Qol visual analog scale (EQ-VAS), which were developed by the EuroQol Group in 2005 (11). The EQ-5D-5L is a generic instrument for describing and evaluating health status by interrogating questions related to five dimensions: mobility (MO), self-care (SC), usual activities (UA), pain/discomfort (PD), and anxiety/depression (AD). Each dimension has five response levels (**Table 1**). The questionnaire is designed for self-completion, has been widely tested in different populations and patient samples, and is routinely used in clinical research (12).

**Table 1:** Comparison of pre- and postoperative EQ-5D-5L dimension levels in the subgroup analysis regarding histopathological features.

		Overall (*n* = 167, *n* = 166 follow-up)^a^	*p*	Group 1 (*n* = 148)	*p*	Group 2 (*n* = 4, *n* = 3 follow-up)^a^	Group 3 (*n* = 15)
EQ-5D-5L dimension level % (*n*)							
Mobility [%, (*n*) with limitations]^b^	T^1^	32.9 (55)	<0.001	29.7 (44)	<0.001	25 (1)	33.3 (5)
	T^2^	22.2 (37)		19.5 (29)		66.6 (2)	13.3 (2)
No problems	T^1^	67 (112)	<0.001	66.8 (99)	0.038	75 (3)	66.6 (10)
	T^2^	77.7 (129)		77.7 (115)		33.3 (1)	86.6 (13)
Slight problems	T^1^	14.9 (25)	0.877	14.8 (22)	1	0 (0)	20 (3)
	T^2^	14,4 (24)		14.8 (22)		33.3 (1)	6.6 (1)
Moderate problems	T^1^	11.9 (20)	0.137	11.4 (17)	0.277	25 (1)	13.3 (2)
	T^2^	7.2 (12)		6.7 (10)		33.3 (1)	6.6 (1)
Severe problems	T^1^	5.3 (9)	0.01	6 (9)	0.01	0 (0)	0 (0)
	T^2^	0.6 (1)		0.6 (1)		0 (0)	0 (0)
Extreme problems/unable to	T^1^	0.6 (1)	0.317	0.6 (1)	0.316	0 (0)	0 (0)
	T^2^	0 (0)		0 (0)		0 (0)	0 (0)
Self-care [%, (*n*) with limitations]^b^	T^1^	17.3 (29)	0.002	14.1 (21)	0.487	25 (1)	33.3 (5)
	T^2^	11.4 (19)		11.4 (17)		0 (0)	13.3 (2)
No problems	T^1^	82.6 (138)	0.192	84.4 (125)	0.308	75 (3)	66.6 (10)
	T^2^	88.5 (147)		88.5 (131)		100 (3)	86.6 (13)
Slight problems	T^1^	10.1 (17)	1	8.1 (12)	0.545	0 (0)	33.3 (5)
	T^2^	10,2 (17)		10.1 (15)		0 (0)	13.3 (2)
Moderate problems	T^1^	4.1 (7)	0.091	4.0 (6)	0.152	25 (1)	0 (0)
	T^2^	1.2 (2)		1.3 (2)		0 (0)	0 (0)
Severe problems	T^1^	2.9 (5)	0.024	3.3 (5)	0.024	0 (0)	0 (0)
	T^2^	0 (0)		0 (0)		0 (0)	0 (0)
Extreme problems/unable to	T^1^	0 (0)		0 (0)		0 (0)	0 (0)
	T^2^	0 (0)		0 (0)		0 (0)	0 (0)
Usual activities [%, (*n*) with limitations]^b^	T^1^	44.3 (74)	<0.001	43.2 (64)	0.03	25 (1)	60 (9)
	T^2^	31.3 (52)		31.0 (46)		33.3 (1)	33.3 (5)
No problems	T^1^	55.6 (93)	0.02	56.7 (84)	0.03	75 (3)	40 (6)
	T^2^	68.6 (114)		68.9 (102)		66.6 (2)	66.6 (10)
Slight problems	T^1^	20.9 (35)	1	20.2 (30)	1	0 (0)	33.3 (5)
	T^2^	21 (35)		20.2 (30)		33.3 (1)	26.6 (4)
Moderate problems	T^1^	19.1 (32)	0.013	18.9 (28)	0.032	0 (0)	26.6 (4)
	T^2^	9.6 (16)		10.1 (15)		0 (0)	6.6 (1)
Severe problems	T^1^	4.1 (7)	0.032	4 (6)	0.056	25 (1)	0 (0)
	T^2^	0.6 (1)		0.6 (1)		0 (0)	0 (0)
Extreme problems/unable to	T^1^	0 (0)		0 (0)		0 (0)	0 (0)
	T^2^	0 (0)		0 (0)		0 (0)	0 (0)
Pain [%, (*n*) with limitations]^b^	T^1^	76 (127)	<0.001	76.3 (113)	<0.001	75 (3)	73.3 (11)
	T^2^	54.8 (91)		54.7 (81)		66.6 (2)	53.3 (8)
No problems	T^1^	23.9 (40)	<0.001	23.6 (35)	<0.001	25 (1)	26.6 (4)
	T^2^	45.1 (75)		45.2 (67)		33.3 (1)	46.6 (7)
Slight problems	T^1^	28.1 (47)	0.06	25.6 (38)	0.018	50 (2)	46.6 (7)
	T^2^	37.9 (63)		38.5 (57)		66.6 (2)	26.6 (4)
Moderate problems	T^1^	23.9 (40)	0.018	26.3 (39)	0.003	0 (0)	6.6 (1)
	T^2^	13.8 (23)		12.8 (19)		0 (0)	26.6 (4)
Severe problems	T^1^	20.9 (35)	<0.001	22.2 (33)	<0.001	0 (0)	13.3 (2)
	T^2^	3 (5)		3.3 (5)		0 (0)	0 (0)
Extreme problems/unable to	T^1^	2.9 (5)	0.024	2 (3)	0.082	25 (1)	6.6 (1)
	T^2^	0 (0)		0 (0)		0 (0)	0 (0)
Anxiety/depression [%, (*n*) with limitations]^b^	T^1^	50.2 (84)	<0.001	52 (77)	<0.001	0 (0)	46.6 (7)
	T^2^	25.9 (43)		26.3 (39)		33.3 (1)	20 (3)
No problems	T^1^	49.7 (83)	<0.001	47.9 (71)	<0.001	100 (4)	53.3 (8)
	T^2^	74 (123)		73.6 (109)		66.6 (2)	80 (12)
Slight problems	T^1^	25.7 (43)	0.046	27 (40)	0.051	0 (0)	20 (3)
	T^2^	16.8 (28)		17.5 (26)		33.3 (1)	6.6 (1)
Moderate problems	T^1^	15.5 (26)	0.009	15.5 (23)	0.016	0 (0)	20 (3)
	T^2^	6.6 (11)		6.7 (10)		0 (0)	6.6 (1)
Severe problems	T^1^	7.7 (13)	0.025	8.1 (12)	0.017	0 (0)	6.6 (1)
	T^2^	2.4 (4)		2 (3)		0 (0)	6.6 (1)
Extreme problems/unable to	T^1^	1.1 (2)	0.156	1.3 (2)	0.156	0 (0)	0 (0)
	T^2^	0 (0)		0 (0)		0 (0)	0 (0)
EQ-5D-%L index (mean +/– SD)	T^1^	0.801 (0.200)	<0.001	0.800 (0.193)	<0.001	0.771 (0.400)	0.818 (0.217)
	T^2^	0.907 (0.113)		0.908 (0.116)		0.879 (0.045)	0.911 (0.094)
EQ-VAS score (mean +/− SD)	T^1^	72.26 (17.73)	<0.001	72.91 (17.73)	<0.001	52.5 (21.016)	71.2 (14.766)
	T^2^	80.77 (15.93		80.98 (16.21)		66.67 (15.275)	81.47 (12.397)

Group 1 (n = 148) represents the benign PNST, group 2 (n = 8) the malignant tumors, and group 3 (n = 15) the rarities. EQ-5D-5L levels were dichotomized into “no limitations” (i.e., level 1) and “with limitations” (i.e., levels 2 to 5).

T^1^, preoperative status; T^2^, postoperative status.

a Missing data due to death (n = 3, follow-up n = 4) and dementia (n = 1).

b EQ-5D-5L dimension responses of any slight, moderate, severe, and extreme problems were grouped into the “with limitations” category.

An index value was calculated based upon the EQ-5D-5L, which reflects the quality of the health status according to the preferences of the general population of a country, in this case, Germany (0: worst possible state of health, 1: best possible state of health). Therefore, the EQ-5D-5L-Crosswalk-Index-Value-Calculator from the research by van Hout et al. was used (13). The EQ-VAS ranges from 0% (worst possible health status) to 100% (best imaginable health status) (11, 12).

Data were collected via face-to-face interviews during follow-up examinations or telephone interviews or completed at home and sent postally.

All in all, the PNTR contains to date 267 patients; for this substudy, the response rate was 64% (*n* = 171). In summary, the surveys were assessed at a mean of 36.9 months (SD 23 months) after surgery.

### Clinical data

Detailed patient characteristics, including age and sex, as well as histopathological diagnosis, type of surgical treatment, neurological symptoms, pain, and imaging-specific data, were analyzed. Surgical therapy was performed according to established principles (14).

### Statistical analysis

Dataset analysis was performed using SPSS 27.0 (SPSS, Inc., Chicago, IL, USA). Metric data were described using median, mean, and standard deviation; categorical data were characterized by frequency and valid percent. Mann–Whitney *U*, Wilcoxon, Fisher exact, McNemar, and chi-square tests were used for the analysis. The correlation was calculated using Pearson correlation. A level of significance was defined as *p <*0.05.

## Results

### Patients’ characteristics

A total of 171 surgically treated patients at three high-volume centers were included in this study. Forty-five percent of patients were women (*n* = 77), and 55% were men (*n* = 94), with a mean patient age of 48.1 years (SD 13.4). Patients were surgically treated by complete tumor resection in 88.9%, by biopsy in 7%, and by partial tumor removal in 4.1%. A neurofibromatosis spectrum disease was scientifically proven in 15 patients (8.7%). The demographic and clinical characteristics of the patients are presented in **Table 2**.

**Table 2:** Patients’ baseline characteristics.

Parameters	Cohort, *n* = 171			
**Follow-up time**	Mean 36.9 (SD 23) months		Range 3–96 months	
**Age**	Mean 48.1 (15–85) years		Median 48 months	
**Sex**	Women 45% (*n* = 77/171)		Men 55% (*n* = 94/171)	
**Side**	Left 51.5% (*n* = 88)	Centrally 1.2% (*n* = 2)		Right 47.4% (*n* = 81)
**Surgical technique**	Gross total resection 88.9% (*n* = 152) Partial resection 4.1% (*n* = 7) Biopsy 7% (*n* = 12)			

Neurofibromatosis spectrum disease 8.7% (n = 15).

### Location

PNSTs were most often located in the brachialis plexus region in 16.4% (*n* = 28), in the median nerve in 15.8% (*n* = 27), and in the ulnar nerve in 12.3% (*n* = 21), respectively. The upper and lower extremities were the location of 96 (56.1%) and 75 (43.9%) tumors. **Table 3** shows the distribution of the 171 peripheral nerve tumors according to their location.

**Table 3:** Location of 171 peripheral nerve tumors.

Location	Percent, frequency
**Upper extremity**	**56.1% (*n* = 96)**
Cervical plexus	1.2% (*n* = 2)
Suprascapularis nerve	1.2% (*n* = 2)
Brachial plexus	16.4% (*n* = 28)
Median nerve	15.8% (*n* = 27)
Ulnar nerve	12.3% (*n* = 21)
Radial nerve	2.9% (*n* = 5)
Cutaneous antebrachii medialis nerve	1.8% (*n* = 3)
Interosseus posterior nerve	1.2% (*n* = 2)
**Lower extremity**	**43.9% (*n* = 75)**
Lumbosacral plexus	4.1% (*n* = 7)
Femoral nerve	5.3% (*n* = 9)
Cutaneous femoral nerve	1.8% (*n* = 3)
Sciatic nerve	10.5% (*n* = 18)
Tibial nerve	7.6% (*n* = 13)
Peroneal nerve	10.5% (*n* = 18)
Saphenous nerve	2.3% (*n* = 4)
**Others (single represented locations)**	5.2% (*n* = 9)

### Histopathology

Most of the 171 surgically treated PNSTs were benign (86.5%) and included schwannoma (*n* = 121) and neurofibroma (*n* = 27). Other histopathological diagnoses were perineurioma (*n* = 6), hybrid nerve sheath tumors (schwannoma/neurofibroma and schwannoma/perineurioma, *n* = 4), and lymphangioma (*n* = 2). Malignant tumors included malignant peripheral nerve sheath tumors (MPNSTs) (*n* = 5). Rarities were singularly represented such as cavernous hemangioma, desmoid tumor, metastasis of breast cancer, amyloidoma, plasmacellular myeloma, and B-cell lymphoma **Table 4**.

**Table 4:** Distribution of histopathological diagnosis within the cohort.

Histopathology	Percent (frequency)
Schwannoma	70.8% (*n* = 121)
Neurofibroma	15.8% (*n* = 27)
Perineurioma	3.5% (*n* = 6)
MPNST	2.9% (*n* = 5)
Hybrid nerve sheath tumor	2.3% (*n* = 4)
Lymphangioma	1.2% (*n* = 2)
Cavernous hemangioma	0.6% (*n* = 1)
Desmoid	0.6% (*n* = 1)
Metastasis of breast cancer	0.6% (*n* = 1)
Amyloid angiopathy	0.6% (*n* = 1)
Plasmacellular myeloma	0.6% (*n* = 1)
B-cell lymphoma	0.6% (*n* = 1)

### Pain

**Figure 1:**
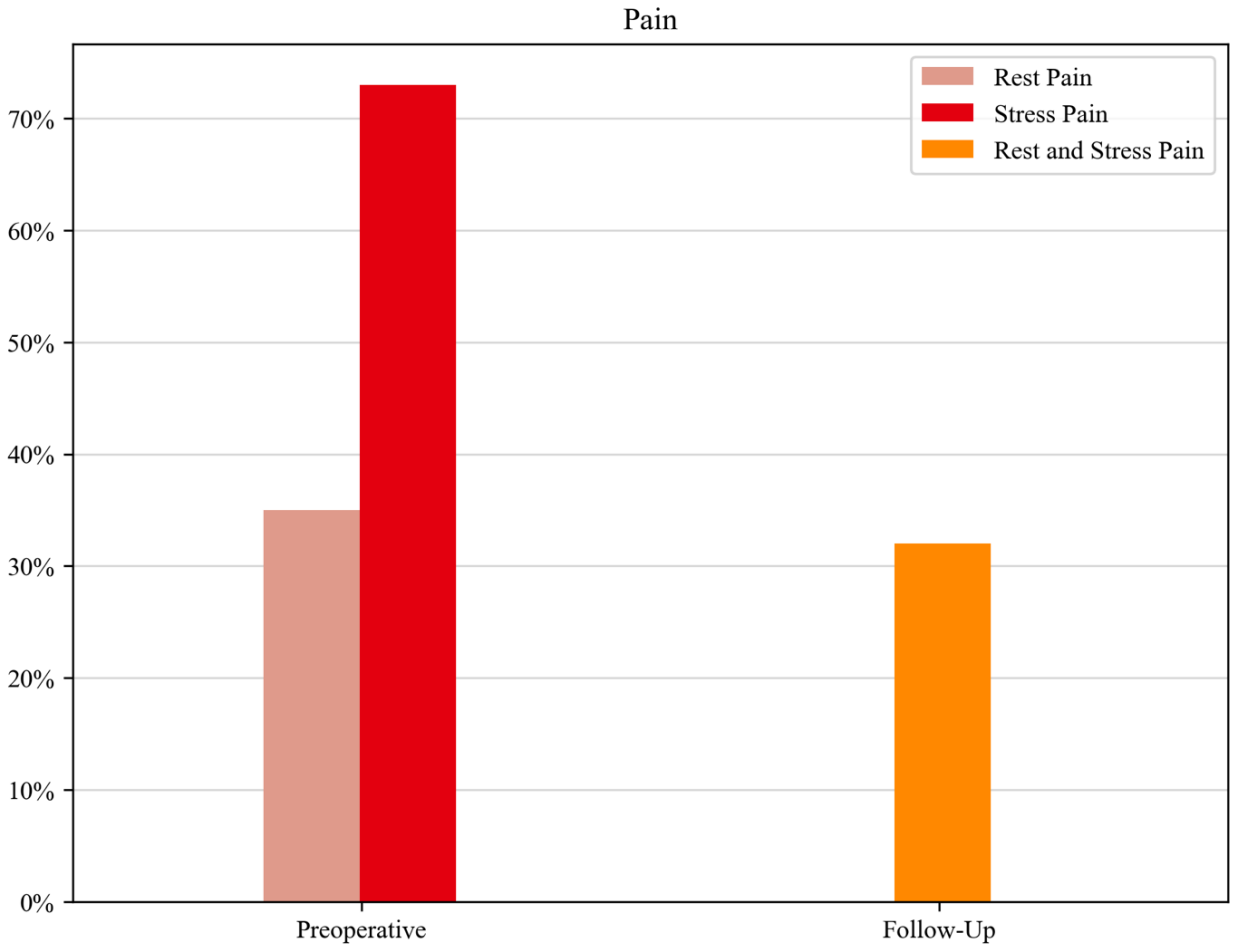
Monitoring of the lead symptom (pain) over the course of the disease.

The prevailing preoperative symptom was pain, including stress and rest pain in 140 patients (82%), rest pain in 60 patients (35%), and stress pain, including a positive Tinel sign in 125 patients (73%). In the follow-up examination, which was in the mean 36.9 months after surgery, patients benefited significantly from surgery, reporting overall pain release (*p* < 0.001). Only 55 patients (32%) reported pain postoperatively (stress and rest pain altogether) **Figure 1**.

### Neurological deficits

**Figure 2:**
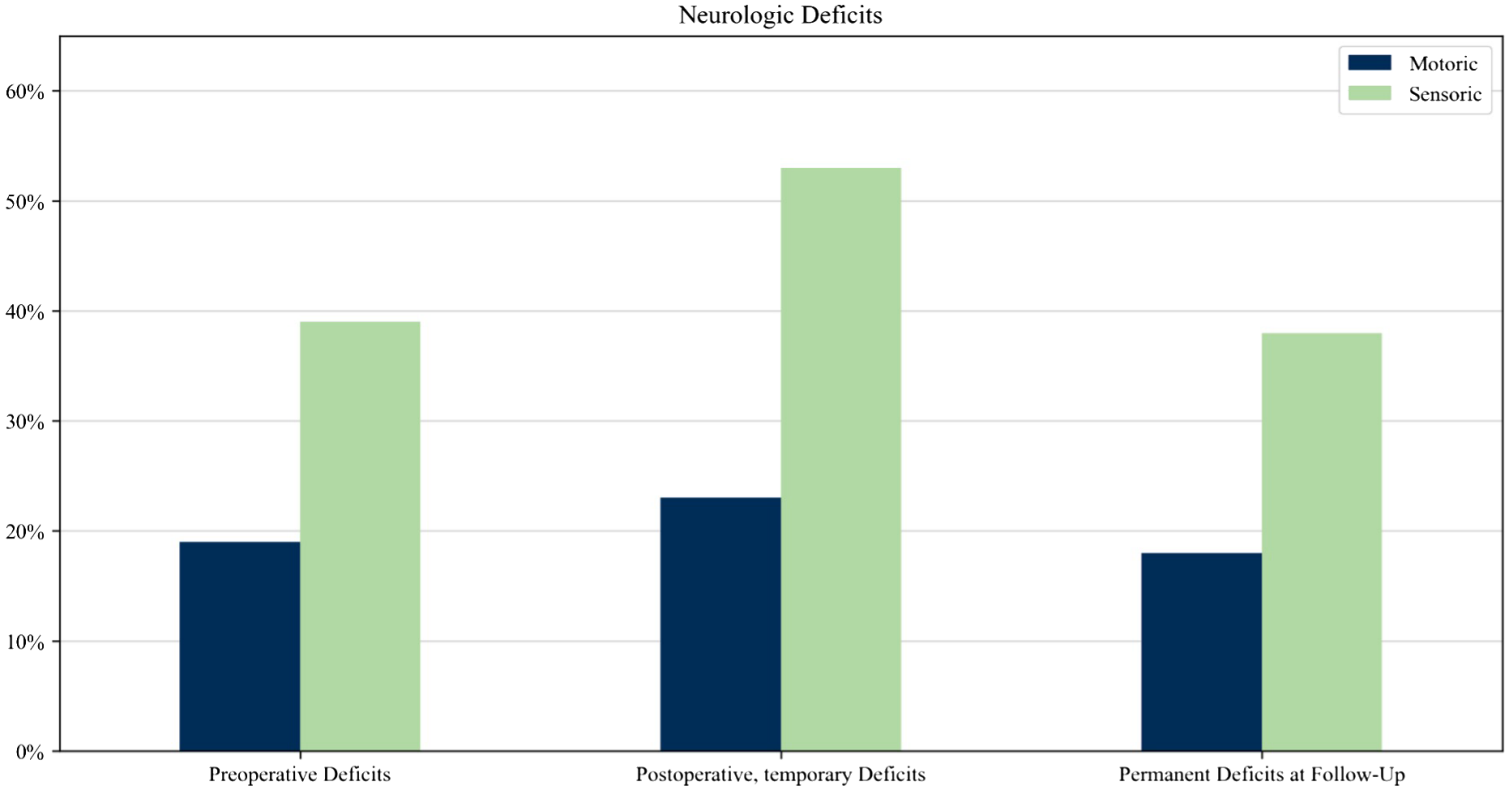
Monitoring of neurological deficits throughout the disease.

Preoperative motor deficits occurred in 18.7% (*n* = 32), which increased postoperatively to 23.4% (*n* = 40) but decreased in the follow-up examination to 18% (*n* = 31) **Figure 2**. In 7 of these 32 patients (21.8%), motor deficits occurred after a previous surgery or biopsy at an unspecialized center.

In the total cohort of 171 patients, 13 patients (7.6%) underwent previous surgery, including biopsies at an unspecialized center. In 7 of 13 patients (53.8%) and in 11 of 13 patients (84.6%), motor deficits and sensory deficits respectively occurred after previous surgery or biopsy at an unspecialized center. Out of 158 patients who had no previous biopsy, 28 patients presented preoperative motor deficits, while 27 cases exhibited deficits. Among the 158 cases, 7 patients (4*%*) experienced new motor deficits after undergoing surgery at a specialized center.

A significant correlation (*p* < 0.001, *r* = 0.744) was found between patients with preoperative motor deficits (*n* = 32) and patients with permanent motor deficits (*n* = 24).

The same trend is seen in sensory deficits. Preoperative sensory deficits occurred in 38.6% (*n* = 66), which increased postoperatively to 52.6% (*n* = 90) but then decreased in the follow-up examination to 38% (*n* = 65). In 11 of these 66 patients (16.6%), sensory deficits occurred after a previous surgery or biopsy at an unspecialized center. We found a weak correlation between the preoperative and postoperative permanent sensory deficit (*p* < 0.001, *r* = 0.443).

### Health-related quality of life data from the Euro-Qol-5D-5L and the Euro-Qol visual analog scale

The median health index value of the entire cohort was 0.887 (*n* = 167) preoperatively and improved postoperatively to 0.910 (*n* = 166, *p* < 0.001). The median EQ-VAS score was 75% (*n* = 167) preoperatively and enhanced to 85% (*n* = 166, *p* < 0.001) postoperatively (**Table 5**).

**Table 5:** Comparison of EQ-5D-5L index values preoperatively (T^1^) vs. postoperatively (T^2^) of the total cohort (*N* = 167).

		Index value T^1^	EQ-VAS score (%) T^1^	Index value T^2^	EQ-VAS score (%) T^2^
***N***	**Valid**	167	167	166	166
	Missing^a^	4	4	5	5
**Mean**		0.801	72.26	0.907	80.77
**Median**		0.887	75.00	0.910	85.00
**Std. deviation**		0.200	17.73	0.113	15.93
**Minimum**		0.118	15	0.378	25
**Maximum**		1.000	100	1.000	100
**Highest score of 1 (EQ-5D-5L) and 100% EQ-VAS, *N* (%)**		21 (12.3%)	9 (5.3%)	57 (33.3%)	19 (11.1%)

T^1^, preoperative status; T^2^, postoperative status.

a Missing data due to death (n = 3, follow-up n = 4) and dementia (n = 1).

EQ-5D-5L levels were dichotomized into “no limitations” (i.e., level 1) and “with limitations” (i.e., levels 2 to 5). The results are shown in **Table 1**.

Preoperatively, 12.3% of the patients reached the highest index value score of 1.0 in the EQ-5D-5L and improved postoperatively to 33.3%. In the Euro-Qol visual analog scale (EQ-VAS score), patients rated their overall health preoperatively with a median of 75%, which improved to 85% postoperatively.

### Subgroup analysis

Health index values and EQ-VAS scores were compared between different subgroups (histopathological diagnosis, age, and gender). According to histopathological features, three subgroups were defined. Group 1 included benign nerve sheath tumors (schwannoma and neurofibroma), with a total of 148 patients. Group 2 included malignant tumors, including MPNST, metastasis of breast cancer, plasmacellular myeloma, and B-cell lymphoma, with a total of eight patients. In group 3, rare histopathological diagnoses were summarized, including perineurioma, hybrid nerve sheath tumors, and lymphangioma, and represented diagnoses with a total number of 15 patients.

Because of the small number of patients in groups 2 and 3, no levels of significance were calculated.

In **Table 1**, all pre- and postoperative EQ-5D-5L dimension levels are compared regarding the distribution in subgroups after histopathological patterns.

In the overall cohort, after surgery, the number of patients with no limitations significantly increased in the dimension’s mobility, usual activities, pain, and anxiety/depression (**Table 1**). Additionally, the EQ-5D-%L index scores and the EQ-VAS scores improved postoperatively significantly (*p* < 0.001) in the overall cohort (**Table 1**).

In the further analysis of our data regarding upper and lower extremities, we did not find any significant differences in the median health index values preoperatively (upper extremity 0.887, lower extremity 0.828) and postoperatively (upper and lower extremity 0.910) and in the median EQ-VAS scores preoperatively (upper extremity 80%, lower extremity 75%) and postoperatively (85%).

## Discussion

Our study is the first to explore the HRQoL of surgically treated patients with peripheral nerve tumors in a multicentric setting.

The EQ-5D-5L questionnaire was able to detect significant differences between pre- and postoperative HRQoL as hypothesized. Consequently, surgery significantly improved the HRQoL as well as individual function in the dimensions, mobility, usual activities, pain, and anxiety in patients with PNT in the entire cohort.

### Comparison of life quality in patients with PNT to the general German population

The mean EQ-5D-5L index score of the German general adult population was stated to be 0.88 (SD 0.18), and the overall EQ-VAS score was 71.59 (SD 21.36). Higher education, full-time work, and private health insurance were associated with a higher EQ-5D-5L index score. Female gender and higher age were associated with a lower EQ-5D-5L index score (12).

In our cohort, the median health index values were identical for the male and female genders (0.887 T^1^, 0.910 T^2^). Women reported a lower score (72.5%) than men (80%) only in the preoperative median EQ-VAS score. Postoperatively, the median EQ-VAS score increased in both genders to 85%. In our cohort, it can therefore be concluded that there was no gender difference in life quality.

In terms of age relevance for HRQoL, we could not find significant differences in our cohort. With a median age of 48 years, our cohort was slightly younger than the cohort described by Grochtdreis et al. with a median age of 51 years (12). Younger age according to their data was associated with better life quality. This correlation could not be found in our dataset of PNT patients.

Overall, patients in our cohort had a higher median index score postoperatively than in the German general adult population (0.91 vs. 0.88). Reduced pain and symptom control could be a possible explanation.

Among the five different dimensions that were analyzed in our cohort, the fewest restrictions were in the dimension of self-care. Nearly all patients can fend for themselves. Disabilities in patients with PNT—excluding NF patients—are mostly limited to one extremity.

### Effect of surgery on pain and neurological deficits on life quality

Our data show overall that patients improved postoperatively in HRQoL. A non-negligible percentage of motor and sensory deficits occurred after previous surgeries or needle biopsies in non-specific centers; those deficits are frequently permanent. The harm that can be caused due to surgical treatment in non-specialized centers has been shown in a recent study and supports our results (4). Overall, the key observation is that patients who presented deficits prior to surgery are more prone to experiencing permanent deficits. Because neurological deficits determine life quality, especially in the domain of mobility, self-care, and usual activities, these previous surgeries can negatively influence HRQoL. Furthermore, due to their rarity, patients often experience a long diagnostic process in the future with the potential of misdiagnosis and severe consequences (44.7%) (4).

Our study shows that preexisting neurological deficits, including pain, are risk factors for permanent deficits. This additionally supports the recommendation to perform surgery at a specialized center to prevent function and consecutively impaired life quality.

In our dataset, most restrictions occurred in the dimension of pain. In 76%, preoperative limitations (slight–extreme problems) occurred, which significantly improved postoperatively to 54.8% (*p* < 0.001). These results are mirrored in the preoperative collected data of symptoms; pain (rest and stress pain) was reported in 82% and improved postoperatively to 32%. All in all, surgery led to improved symptom control in the dimension of pain. Pain was not only the leading symptom in patients with malignant or rare PNTs but also in patients with benign PNTs like schwannoma and neurofibroma with no other neurological deficits, which is in concordance with previous studies (2, 15). For this reason, we recommend early surgery at a specialized center for symptom control and for returning to everyday life and work quickly.

### Effect of histopathological diagnosis on life quality

Compared with patients with benign PNT, patients with malignant peripheral nerve tumors undoubtedly have a worse life quality (**Table 1**), which is, on the one hand, related to the physio- and psychological burden of the disease itself and, on the other hand, influenced by the more radical surgical treatment followed by neurological deficits and the duration of the disease. Due to the small number of patients with malignant PNT, generalized recommendations are not possible; however, due to limited life expectancy, it is even more critical to counterbalance the radicality of treatment and the QoL. Different concepts regarding the priority of neurological function preservation in contrast to surgical radicality are currently applied and must be discussed with the patient before surgery (16). Interestingly, patients in the group with rare entities had comparable pre- and postoperative index scores to the group with benign PNT (T^1^ 0.818, T^2^ 0.911).

The effect that the degree of HRQoL improvement varies according to preoperative neurological symptoms is not only shown in our data but also by Haider et al., who report this effect in patients with intracranial meningiomas (17).

### Socioeconomic aspects of life quality in the working population

As diagnosis and treatment modalities have improved over time, early return to work and life quality are necessary outcome measurements in patients with PNT. Additional work and employment are essential factors in life quality, ranked right after family and partnership (18). It becomes clear that PNT concerns, in particular, the working population with a mean age of 48.1 years. With pain as the leading symptom and knowing the obstacles and the effort that occur in patients with chronic pain returning to work, including managing symptom control, work relationships, and making workspace adjustments (19), the highest goal should be treating pain immediately at the initial diagnosis. The more neurological deficits occur pre- or postoperatively, the more likely it may be that patients will not return to work as fast as patients without deficits. However, a specific analysis of return to work in patients with PNT needs to be improved. Our data show that resection at a specialist high-volume center is safe and improves symptoms and life quality after surgery.

### Strengths and limitations

The strength of this study is the large cohort of patients with clearly peripheral nerve tumors; intraspinal schwannoma was not included. A second strength is the multicentric study design.

The limitations are that the results are from a retrospective analysis. The number of patients with malignant PNT and neurofibromatosis was proportionally small.

## Conclusion

Preservation and, if possible, improvement of neurological function and reduction of pain are of utmost importance for individual patient’s quality of life and in patients with PNT. The dimension of pain predominantly affected the overall quality of life. In summary, surgical therapy improved life quality in the entire cohort.

## Data availability statement

The original contributions presented in the study are included in the article/supplementary material. Further inquiries can be directed to the corresponding author.

## Ethics statement

The studies involving humans were approved by Ethikkomission der Universität Ulm. The studies were conducted in accordance with the local legislation and institutional requirements. Written informed consent for participation in this study was provided by the participants’ legal guardians/next of kin.

## Author contributions

NG: Conceptualization, Data curation, Formal analysis, Investigation, Methodology, Validation, Visualization, Writing – original draft. UAK: Data curation, Writing – review & editing. CW: Supervision, Writing – review & editing. ND: Conceptualization, Data curation, Supervision, Writing – review & editing. RK: Conceptualization, Methodology, Supervision, Writing – review & editing. AP: Conceptualization, Formal analysis, Project administration, Resources, Visualization, Writing – original draft, Writing – review & editing. GA: Conceptualization, Supervision, Writing – review & editing. BM: Formal analysis, Methodology, Writing – review & editing. MP: Conceptualization, Data curation, Methodology, Supervision, Validation, Writing – original draft, Writing – review & editing.
